# Developing a smart and scalable tool for histopathological education—PATe 2.0

**DOI:** 10.1016/j.jpi.2025.100535

**Published:** 2025-12-05

**Authors:** Lina Winter, Annalena Artinger, Hendrik Böck, Vignesh Ramakrishnan, Bruno Reible, Jan Albin, Peter J. Schüffler, Georgios Raptis, Christoph Brochhausen

**Affiliations:** a Institute of Pathology, University Medical Centre Mannheim, Medical Faculty Mannheim, Heidelberg University, Mannheim, Germany; b Institute of Pathology, University Regensburg, Regensburg, Germany; c Institute of Pathology, TUM School of Medicine and Health, Technical University of Munich, Munich, Germany; d eHealth Laboratory, OTH Regensburg, Regensburg, Germany

**Keywords:** Virtual microscope, Digital pathology, Medical education, Scalability, Microservices architecture, Whole-slide imaging (WSI), User-centric design, Interactive learning, Artificial intelligence integration

## Abstract

Digital microscopy plays a crucial role in pathology education, providing scalable and standardized access to learning resources. In response, we present PATe 2.0, a scalable redeveloped web-application of the former PATe system from 2015. PATe 2.0 was developed using an agile, iterative process and built on a microservices architecture to ensure modularity, scalability, and reliability. It integrates a modern web-based user interface optimized for desktop and tablet use and automates key workflows such as whole-slide image uploads and processing. Performance tests demonstrated that PATe 2.0 significantly reduces tile request times compared to PATe, despite handling larger tiles. The platform supports open formats like DICOM and OpenSlide, enhancing its interoperability and adaptability across institutions. PATe 2.0 represents a robust digital microscopy solution in pathology education enhancing usability, performance, and flexibility. Its design enables future integration of research algorithms and highlights it as a pivotal tool for advancing pathology education and research.

## Introduction

Pathology education is undergoing a transformative shift, as digital technologies replace traditional microscopy and significantly influence how medical students learn.^1^^,^^2^ Developments in robotics and artificial intelligence (AI) are changing medical practice and emphasize the growing need for integrating digital solutions into educational environments.^3^ Conventional microscopy is dependent on small group access to physical slides. It faces challenges of variability, logistical constraints, and limited scalability, which are all inadequate for the needs of modern students.^4^^,^^5^ Conversely, digital microscopy offers many advantages. For instance, modern platforms provide access to high-quality, standardized slides, eliminating the variability and logistical limitations of physical slide handling. This allows students to review material independently on personal devices, which leads to higher flexibility and self-directed learning. Additionally, digital platforms integrate seamlessly with e-learning tools, incorporating annotation tools, interactive quizzes, multimedia resources, and collaborative features that enhance engagement and comprehension.^5^^,^^6^ In pathology education, the demand for digital solutions is well studied. Rodrigues-Fernandes et al. systematically reviewed that digital microscopy performs equal or superior to traditional methods in improving student outcomes.^7^ Correspondingly, Brochhausen et al. further reported a high demand for digital learning tools. Specifically, 91.9% of students were using these resources, primarily for self-study.^4^ During the COVID-19 pandemic, the need for scalable, accessible learning systems was further emphasized when universities and students faced periods of remote learning.^8^, ^9^, ^10^

However, current systems for digital pathology education often fall short in meeting these needs. Centralized slide servers require complex configurations, such as VPN access, and often rely on proprietary viewer applications that lack broader compatibility.^11^^,^^12^ Similarly, cloud-based solutions, while eliminating the need for local software, lock institutions into vendor ecosystems, limiting flexibility and scalability. Both approaches struggle to address the growing demand for collaborative, open-standard platforms that can integrate research algorithms and meet diverse institutional needs.

PATe 2.0 is a web-based continuation and redevelopment of the original PATe system introduced by Brochhausen et al. in 2015. It overcomes the limitations of the previous system by using the OpenSlide library to support all major slide formats, including DICOM. Unlike traditional systems, PATe 2.0 is hardware-agnostic and OS-independent which enables institutions to utilize existing resources while fostering interoperability between universities and research centers. Its modular design supports research integration and allows custom algorithms to be seamlessly embedded into the platform. Therefore, PATe 2.0 does not only represent an effective educational resource but also a bridge between academia and current developments in pathology research.

The present report describes the redevelopment of the PATe system into PATe 2.0, a smart and scalable tool for histopathological education. Our web-based platform aims to offer non-restrictive access to a virtual microscopy education platform in which the students can zoom in and out smoothly and can move within the whole-slide image (WSI) continuously. It addresses key limitations of the original platform that led to the collapse of its virtual machine (VM) and improves its scalability, user interface, and system integration. This updated version offers a practical contribution to the ongoing digitization of pathology education.

## Methods

The iterative development process and project planning of PATe 2.0 followed an agile methodology, coupled with a systematic version control system, to ensure flexibility and responsiveness to changing requirements. The approach enabled efficient feedback loops, continuous improvements, and effective stakeholder engagement.

### Agile development process

The agile methodology was chosen for its adaptability to evolving project needs, unlike the rigid waterfall model that requires predefined requirements.^13^ The lack of documentation and knowledge about the original PATe system prevented the identification or the formulation of requirements for its extension and repair. The decision to completely redesign PATe into PATe 2.0 further validated this approach, allowing iterative adjustments and stakeholder feedback to guide development effectively.

### Version control with Git

Git was used as the primary version control system, providing capabilities for tracking changes, reverting to previous iterations, and managing multiple development branches.^14^ The centralized organization of the Git repository enabled integration with deployment pipelines, facilitating automated deployments within the microservices architecture. This approach ensured consistency and reliability during the iterative development process.

### Microservice architecture

Microservice architecture is an approach to develop a single application as a series of small, independent services, each running in its own process and communicating via Application Programming Interfaces (APIs).^15^ These services can be provided independently of each other by fully automated provisioning engines. There is minimal centralized management of these services, which may be written in different programming languages and use different data storage technologies.

### Iterative development framework

**Fig. 1:**
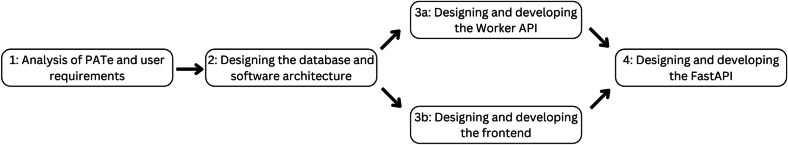
Development of a workflow for the PATe2.0 platform.

The development process, shown in Fig. 1, was organized into five distinct iterations. At the end of each iteration, meetings were held with the project stakeholders to present progress, gather feedback, and refine requirements.

#### Iteration 1: Analysis of PATe and user requirements

The initial iteration involved analyzing the existing PATe codebase to identify its limitations and potential causes for the system's failure. This analysis revealed the necessity of a complete reimplementation of PATe. A detailed report on PATe's problems and deficiencies was created, forming the foundation for the redesign and redevelopment into PATe 2.0. The scope of the new implementation, along with additional requirements and expected features, was established in collaboration with the stakeholder. Based on this feedback, the iteration plan was revised to accommodate the development of PATe 2.0.

#### Iteration 2: Designing the database and software architecture

In the second iteration, a suitable software architecture for PATe 2.0 was designed and finalized. A microservices architecture was selected for its scalability, modularity, and ease of integration.^16^ A database model was also created to support the platform's current and future requirements. This model emphasized flexibility to allow for subsequent enhancements and iterations.

#### Iteration 3a: Designing and developing the worker API

The third iteration focused on the Worker API's design and implementation. Python 3.11 was chosen as the programming language for its extensive library support and suitability for handling computationally intensive tasks. The Worker API formed the foundation of the backend, providing core functionalities such as slide processing and annotation handling. The iteration concluded with a fully functional backend.

#### Iteration 3b: Designing and developing the frontend

The fourth iteration involved the design and implementation of the frontend using the React JavaScript library. It was selected for its responsiveness and compatibility with Material UI and Elastic UI. This framework supports an intuitive, modern interface optimized for use on multiple devices, including tablets and desktops.

#### Iteration 4: Designing and developing the Fast API

The final iteration aimed to optimize the virtual microscopy experience by designing and implementing the Fast API. Developed in the Go programming language, the Fast API was tailored to minimize REST request times for tile loading within WSIs.^17^ This iteration concluded with the presentation of the PATe 2.0 prototype, showcasing its fully integrated features and improved performance.

### Clustering and performance testing

To finally evaluate performance, both PATe and PATe 2.0 were deployed under controlled and comparable conditions.

PATe was deployed as a monolithic application on a Linux server running Ubuntu 18.04. The database was integrated into the same server, and tiles were processed with a size of 256 × 256 pixels, returned as JPEG images.

PATe 2.0 was deployed using Docker containers on a Linux server running Ubuntu 22.04. The deployment consisted of five containers: Worker API, Fast API, Proxy, Database, and Frontend. Tile size was set to 512 × 512 pixels. Load balancing was intentionally disabled to allow a direct comparison between the microservices architecture of PATe 2.0 and the monolithic design of PATe.

Both deployments were run on identical VMs, configured with 4 vCPUs, 10GB RAM, and a dedicated 1 Gbit/s network interface to the test client. The average ping time between the client computer and the server was 0.467 ms, which was subtracted from the measured request times to ensure accuracy. Performance testing was conducted using the open source k6 tool from Grafana Labs.^18^ The test simulated 10 virtual users, each requesting the same tile 10,000 times to simulate active usage scenarios. The average, minimum, and maximum request times were recorded and visualized using error bars to indicate performance variability.

## Results

The implementation of PATe 2.0 has demonstrated substantial improvements in scalability, usability, and functionality compared to its predecessor and competing solutions.

### Iteration 1: Analysis of PATe and user requirements

The first iteration analyzed the PATe system to identify its limitations and determine the feasibility of reusing its codebase. PATe, launched in 2015 and disconnected in 2021, failed due to critical issues, including the collapse of its VM. The analysis revealed several major problems that rendered the codebase unsuitable for further use. The absence of in-code and external documentation, coupled with the original developer's departure, made maintenance and error resolution impossible. Additionally, PATe's monolithic architecture caused high interdependence among components, complicating feature integration and maintenance. Its vertical scalability was insufficient for increasing user loads, and load balancing was impractical. Hardcoded strings for server addresses and credentials were embedded in the code, creating security vulnerabilities, and requiring code changes for updates. Furthermore, PATe was depended on the proprietary Zoomify format, which restricted compatibility to specific scanners and required manual file conversion, significantly limiting efficiency and scalability. Lastly, delays in tile rendering during zooming disrupted the user experience, preventing the immersive microscopy simulation PATe aimed to achieve. The analysis took one working week.

### Iteration 2: Design of architecture and database

**Fig. 2:**
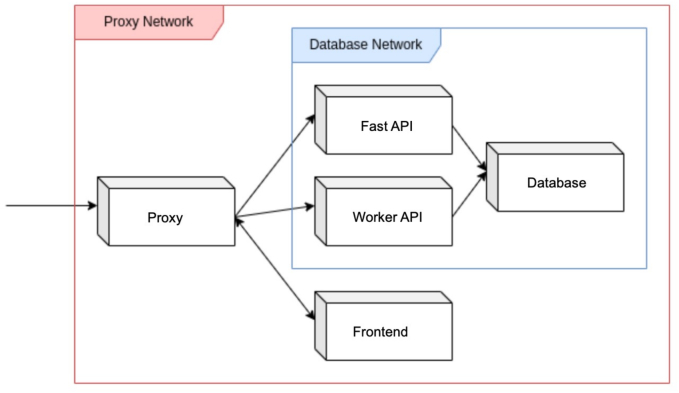
Microservices architecture of PATe 2.0 with a modular structure, including the proxy network, APIs, frontend, and database services.

The second iteration focused on designing the architecture and database to support the scalability and robustness of the new PATe 2.0 system. A microservices architecture was selected to ensure loose coupling between components, enabling independent updates and deployments. This approach also simplified error identification and elimination, as individual services could be scaled horizontally to meet various demands. Five core services, illustrated in Fig. 2, were specified and developed, creating a foundation for integrating internal research algorithms.

A proxy service was implemented to handle communication between the web-application and APIs, acting as a load balancer for scaled containers. It also managed SSL encryption for secure network communication, reducing complexity in the APIs by allowing them to operate solely with the HTTP standard. Envoy was chosen for its low overhead, detailed logging capabilities, and optimization for microservice meshes.

The frontend was developed to provide a web-based user interface, requesting and processing data through the APIs without embedding program logic. This separation of frontend and backend ensures the interface can be easily extended and modified.

The backend was divided into two logical services: the Worker API and the Fast API. The Fast API was optimized for high-frequency, low-complexity operations, whereas the Worker API handled complex, resource-intensive tasks such as slide conversion and importation. This division met the performance and integration requirements established in the analysis phase.

**Fig. 3:**
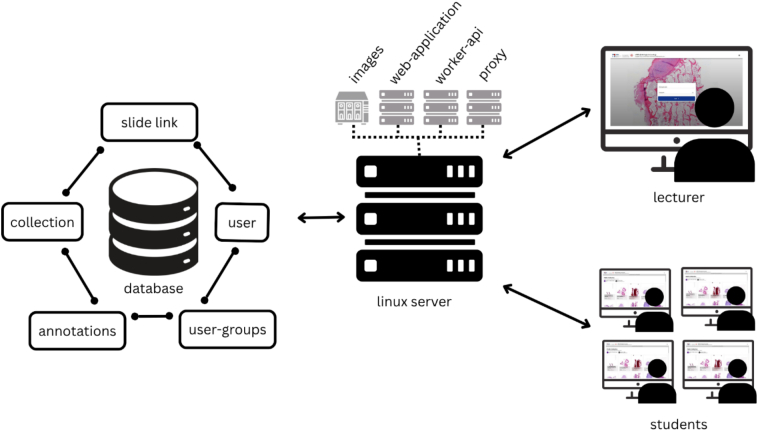
Architecture of PATe 2.0 with a virtualized server structure, a NAS system for image storage, and the database containing user, group, annotations, and image link information.

The overall architecture is illustrated in Fig. 3.

**Fig. 4:**
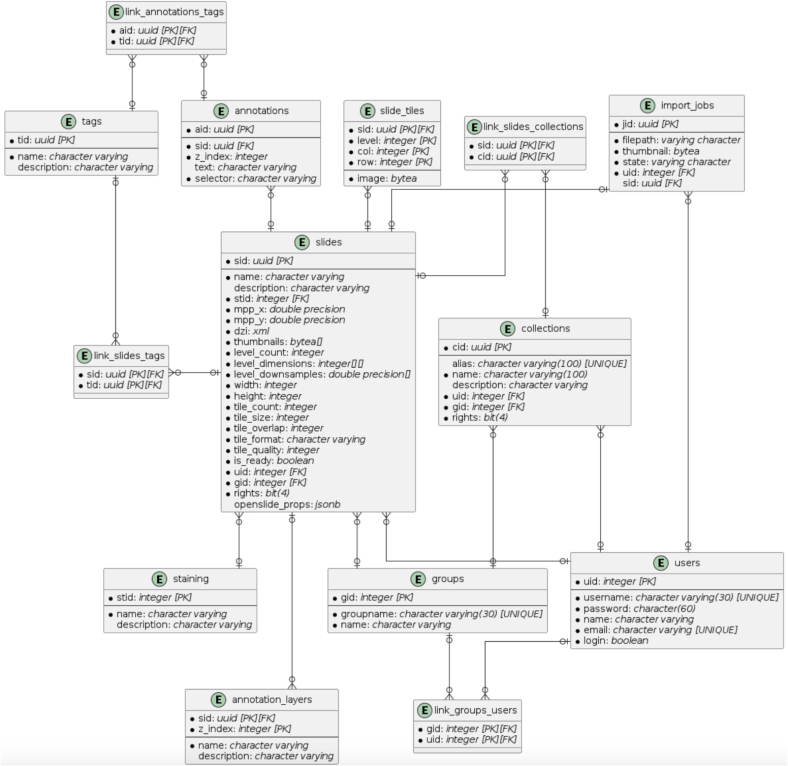
Entity-relationship model of the PATe 2.0 database illustrating the relational structure, including key tables, relationships, and metadata storage.

A relational database model was chosen to store user data, WSIs, and management data Fig. 4. PostgreSQL was implemented for its extensive support for data types such as XML and JSON, allowing efficient storage and querying of metadata captured by OpenSlide. JSON metadata was stored in the openslide_props field of the slides table for research and analysis purposes. To enhance security, user passwords were hashed using the pgcrypto extension.

The database design supported efficient retrieval of thumbnails and WSIs, with fields optimized for mobile device queries. For instance, the thumbnails field in the slides table used the bytes[] type to store multiple resolutions for faster access. User management takes place via the assignment of user groups and the rules contained therein. Structures for supporting annotations and tags were included in the entity-relationship model but deferred for future iterations. To address high access demands on the slide_tiles table, which stores individual tiles of WSIs, future iterations plan to integrate an in-memory database like Redis to further reduce query times during simultaneous user access. The software design and database structuring took 1.5 working weeks.

### Iteration 3: Designing and developing the Worker API

The third iteration focused on implementing the Worker API to handle computationally intensive tasks such as WSI processing and the integration of machine learning algorithms. Python 3.11 was chosen for its compatibility with OpenSlide and its seamless integration with internal AI algorithms developed at our institute.^19^^,^^20^ The Worker API was implemented as a modular RESTful service using the internally developed *raptor* library, which extends Flask with enhanced logging and regex-based routing for more complex API paths.

**Fig. 5:**
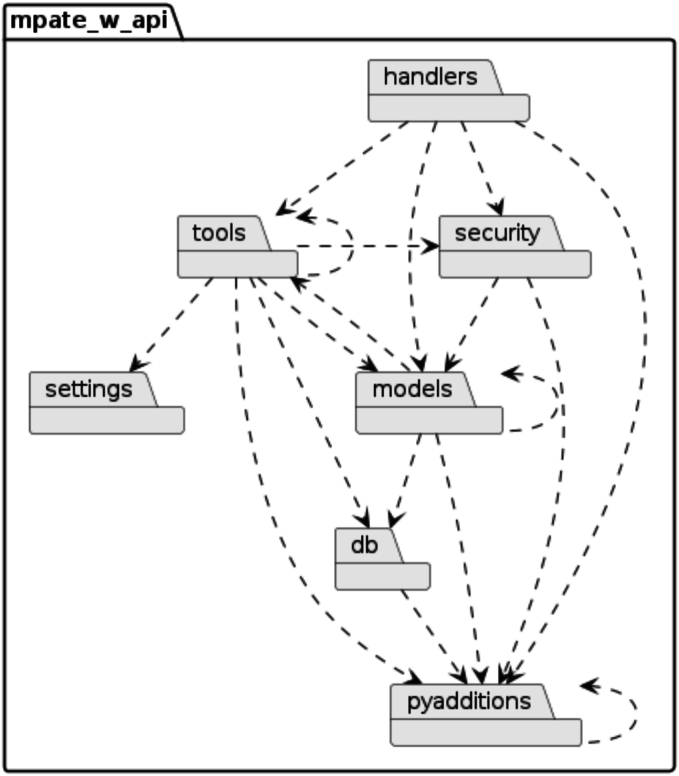
Package diagram of the Worker API illustrating the modular structure of the seven core subpackages and their interdependencies.

As shown in Fig. 5, the Worker API was divided into seven core subpackages:

1. Handlers: Processes REST API requests, with each API path linked to a specific function that ensures type-safe parameters and Flask-based responses.
2. Tools: Provides utilities for WSI preprocessing, such as thumbnail generation, tile extraction, and import job creation.
3. Security: Implements JWT-based authentication for user access and slide permissions, ensuring secure API interactions.
4. Settings: Manages configurable variables from files, command-line arguments, and environment variables, providing flexibility for deployment.
5. Models: Maps the database structure to Python classes, enabling efficient interactions with PostgreSQL. Features such as on-demand loading and serialization reduce bandwidth and query times.
6. db: Manages database connections using a singleton pattern and converts Python data structures into PostgreSQL-compatible formats.
7. pyadditions: Introduces custom classes like Result<T > and Option<T > to enhance error handling, reduce try-catch statements, and improve maintainability.

This modular structure ensures the Worker API is efficient, maintainable, and scalable. For instance, the caching mechanisms implemented in the *pyadditions* package reduce redundant database queries, and the serialization features in *models* enable flexible data outputs. By separating responsibilities across the seven packages, the Worker API simplifies future extensions and promotes clean code practices. The implementation of the worker-API required four working weeks.

### Iteration 4: Design and implementation of the frontend

The fourth iteration focused on designing and implementing the PATe 2.0 frontend to deliver a user-friendly and accessible interface. The React JavaScript library, combined with Material UI and Elastic UI, was chosen for its flexibility and modern design components. OpenSeadragon was integrated as the image rendering engine to enable smooth navigation and interaction with WSIs. Usability on tablets was a key focus, ensuring seamless touch navigation without compromising information clarity. The interface was compiled into a static HTML webpage and containerized using an Apache web server for deployment. The design prioritized simplicity and accessibility to meet the needs of educators and students, avoiding unnecessary multimedia elements that could overwhelm users. The interface features clear navigation, legible typography, and a high text-to-background contrast ratio of 21:1, improving readability and accessibility.

**Fig. 6:**
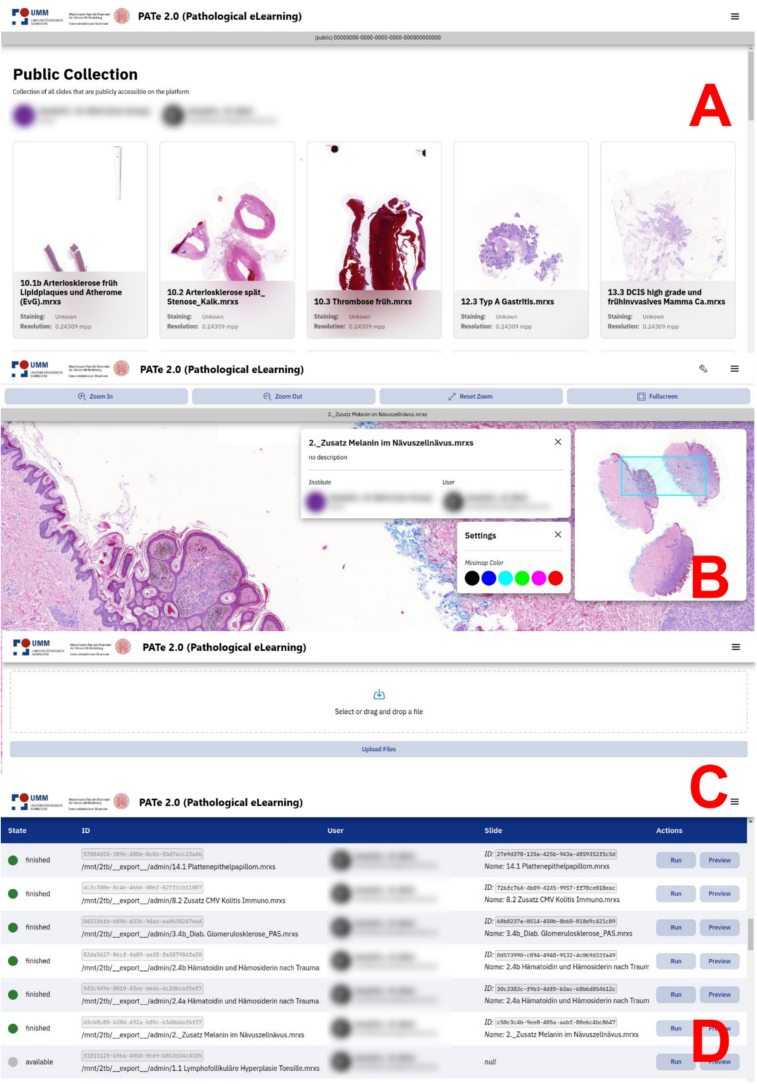
Screenshots of the PATe 2.0 frontend interface with a focus on usability, accessibility, and simplicity. (A) Public Collection interface for selecting WSIs, (B) WSI viewer with tools and settings, (C) WSI upload interface, and (D) Importer interface for managing uploaded WSIs. The implementation of the frontend takes around three working weeks.

As illustrated in Fig. 6, the PATe 2.0 interface remains clean and intuitive to support effective learning. The focus was set on a structured layout of the public collection interface, where users can effortlessly select WSIs and display essential tools on demand. Additional information about WSIs can be accessed when needed, enhancing the user experience.

The process for importing WSIs was significantly simplified compared to PATe. Previously, manual conversion and FTP-based uploads required technical expertise, posing a barrier for educators. In PATe 2.0, WSIs can be uploaded directly through a graphical interface (Fig. 5C), and the import process is initiated via the Importer interface (Fig. 5D) with a single click. This streamlined approach eliminates the need for command-line tools and enables educators to manage WSIs independently.

### Iteration 5: Design and implementation of the Fast API

The fifth iteration focused on implementing the Fast API to efficiently manage high-frequency, low-complexity REST requests. The Fast API, written in Go, was developed to provide fast and reliable responses for operations such as tile loading and user queries.

**Fig. 7:**
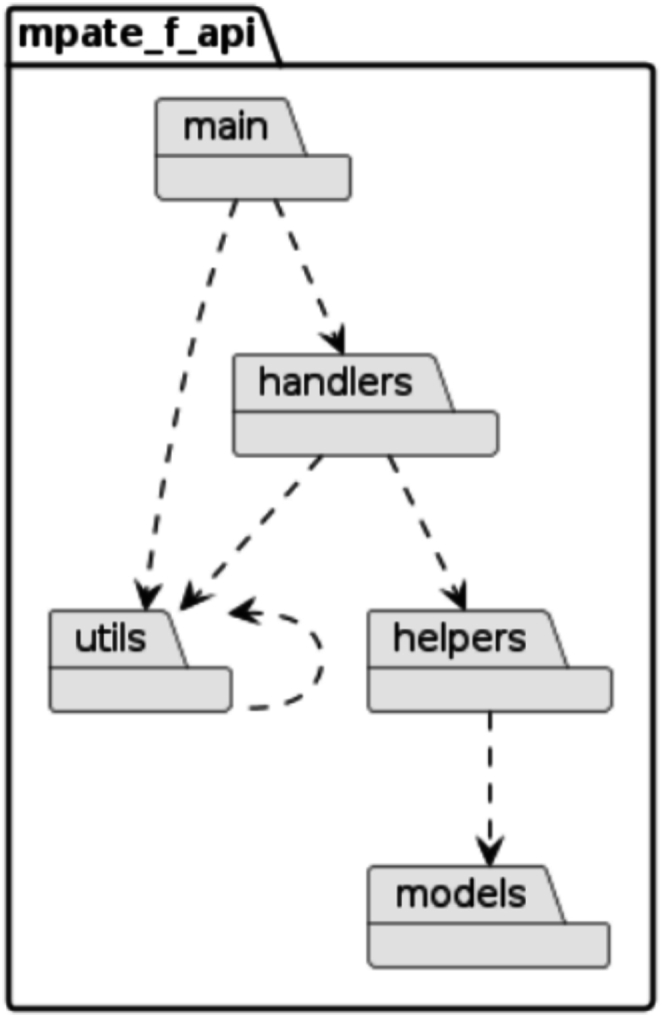
Package diagram of the Fast API displaying the core packages (main, handlers, utils, helpers, and models) and their relationships within the Fast API.

The Fast API is structured into the following core packages (Fig. 7):

1. Handlers: Handles individual REST requests, processing them into logical queries before returning serialized responses.
2. Utils: Manages database connections, configurations, and command-line overrides, ensuring flexibility and adaptability for containerized deployments.
3. Helpers: Executes parameterized database queries and processes logical requests passed from the handlers package.
4. Models: Defines JSON-serializable classes that represent the database structure and simplify data exchange.
5. Main: Initializes the API application, assigns API routes, and starts the HTTP server.

The modular design ensures the Fast API is highly maintainable and easily extendable. The handlers package simplifies REST request handling, whereas the utils centralizes configuration management, enabling rapid adjustments without modifying core files. The helpers package separates business logic from REST-specific functions, improving code clarity and maintainability. Additionally, the models package ensures clean serialization of database objects into JSON responses.

This structure enhances system performance, reduces latency, and ensures smooth communication with the frontend.

### Performance comparison

**Fig. 8:**
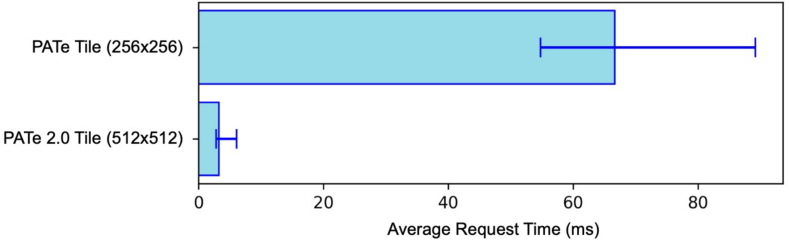
Comparison of average request times for tile loading in PATe and PATe 2.0. Despite using larger tiles, PATe 2.0 demonstrates faster and more consistent performance.

To evaluate the efficiency of PATe 2.0's microservices architecture, a performance test was conducted against PATe using identical conditions, for example, same server hardware, Linux distribution, or network speed. Whereas PATe processed tiles of 256 × 256 pixels, PATe 2.0 processed larger tiles of 512 × 512 pixels. Despite the larger size, PATe 2.0 achieved an average request time of 4 ms (min: 3 ms, max: 7 ms), compared to 66 ms for PATe (min: 54 ms, max: 89 ms). The results are visualized in Fig. 8.

This significant improvement highlights the benefits of PATe 2.0's modular architecture, optimized APIs, and improved database design. Even when factoring in tile size, PATe 2.0 demonstrates faster and more stable performance, enabling a seamless user experience in digital microscopy. The implementation of the fast-API required 3 weeks of work.

## Discussion

Here, we presented the setup of the PATe 2.0, a scalable, hardware-agnostic digital microscopy platform. Key innovations include the user-centric design, the support for diverse slide formats, and enhanced performance through microservices. Furthermore, PATe 2.0's modular design supports the integration of machine learning algorithms, allowing automated analysis of WSIs for research purposes. This functionality promotes collaboration and innovation, bridging the gap between research and education.

There exist several other histological e-learning platforms. However, most of the scientific literature focuses on evaluating the learning efficiency of e-learning platforms and technical background information is rarely reported.^21^, ^22^, ^23^ For instance, CytoAcademy, a web-and mobile-based e-learning platform, has been launched by the Korean Society for Cytopathology. This platform includes an image atlas with descriptions of specific diseases, video lecture material, more than 4000 WSIs, and a quiz section.^23^

PathoDiscovery represents another interactive web-based educational tool of the Department of Pathology in Nijmegen,^24^ the Netherlands. Interestingly, Buma et al. give insights in the technical architecture of PathoDiscovery. Specifically, PathoDiscovery is developed with the Laravel framework and uses PHP and JavaScript enabling users to create learning content easily without programming skills. It integrates with the Pathomation WSI-viewer for handling histological images and connects with educational platforms like Brightspace using LTI links, focusing on user-friendliness and straightforward integration.^24^ In contrast, PATe 2.0 applies a microservices architecture using Go and Python, offering robust scalability and customization. Its frontend is built with React.js, providing a dynamic user experience, and it likely manages digital slides internally, offering more control over image processing functionalities. Thus, whereas PathoDiscovery delivers easy setup and integration, PATe 2.0 appeals to users requiring a high degree of customization and scalability in their educational tools.

Furthermore, VM3.0 represents another educational digital slide platform and was developed under the Erasmus+ program involving multiple European universities.^25^ VM3.0 and PATe 2.0 differ significantly in their technical infrastructure and collaboration models. VM3.0 utilizes a centralized hosting approach with uniform technology across all partner universities, supporting modern technologies like PHP, Node.js, and MariaDB, and specialized libraries for high-performance image processing. This setup facilitates a collaborative network that supports shared access to a virtual microscopy slide library. Thus, VM3.0 exemplifies how a collaborative network can be effectively established, potentially serving as a model for PATe 2.0 when considering expansion to include connectivity with other universities.^25^

The implementation of PATe 2.0 in pathology seminars at the Medical Faculty Mannheim of the Heidelberg University will be finalized by the end of 2025. To ensure a smooth transition, the platform will first be introduced in doctoral seminars, allowing educators to familiarize themselves with the features. Furthermore, this phase will help to identify and address potential issues early on. The implementation process will be evaluated through structured surveys of both medical students and educators, collecting feedback on usability, functionality, and overall effectiveness to guide potential improvements.

Due to its microservice architecture, PATe 2.0 offers enormous development potential. Future iterations of PATe 2.0 will include a quiz or exam mode that supports multiple quizzes and offers different question types such as interactive features such as WSI annotations or naming specific structures. This progress is designed to provide a comprehensive self-training system that empowers students to actively engage with the histological teaching material. Because feedback is essential in online learning, explicit feedback options and self-assessment tools are crucial to help students to identify knowledge gaps and compare their level of knowledge relative to their fellow students.^26^ Furthermore, educators may benefit from PATe 2.0 regarding the efficient generation of tests. In this context, they could use the quiz mode to assess the complexity of questions based on student response accuracy and time and plan sufficient time for test items in the exam situation. Therefore, PATe 2.0 might support educators to find a balance between excessive demand and underload among the students and to finally ensure educational success.

Additionally, an advanced search function is planned, enabling users to filter WSIs and annotations more effectively, thereby enhancing both teaching and research applications. Whereas PATe 2.0 was developed primarily for educational purposes, its flexible and scalable architecture enables applications beyond the classroom. In multidisciplinary team meetings, for example, PATe 2.0 can facilitate real-time collaboration by providing simultaneous access to high-resolution WSIs, allowing pathologists, radiologists, and clinicians to discuss complex cases efficiently.^27^ This capability might enhance diagnostic accuracy and support more informed therapy decisions. In telemedicine, PATe 2.0 aligns with the growing demand for remote diagnostic capabilities, providing a robust platform for telepathology consultations. Haghighi et al. demonstrated that transitioning from physical slides to digital WSIs improves efficiency, diagnostic accuracy, and safety by eliminating logistical challenges associated with physical slide handling. Notably, the digital transition significantly improved time efficiency, reducing the consultation turnaround time from 86 h to an average of just 35 min.^28^ With PATe 2.0, healthcare institutions might expand access to pathology expertise, particularly in underserved or remote regions, bridging gaps in healthcare quality and equity.^29^^,^^30^ By taking advantage of its open and modular design, PATe 2.0 might be integrated into existing telehealth systems, enabling a scalable, cost-effective solution in digital pathology. Furthermore, the integration of machine learning algorithms into PATe 2.0 aligns with the growing trend of utilizing AI in pathology for diverse image analysis and segmentation tasks.^31^^,^^32^ These applications range from fundamental tasks, such as the identification and classification of cells, to more advanced capabilities, including image pattern recognition to predict disease diagnoses, assess prognostic indicators, and guide therapeutic strategies.^33^ By bridging education, diagnostics, and research, PATe 2.0 demonstrates its potential as a flexible tool for advancing pathology in clinical and multidisciplinary contexts.

However, the implementation of PATe 2.0 poses certain challenges that should be addressed to guarantee its broader adoption and effectiveness. Deploying a microservices architecture, while offering scalability and modularity, requires significant technical expertise in containerization, orchestration (e.g., Kubernetes or Docker Swarm), and network configuration. Smaller institutions, particularly those with limited IT resources, may find it challenging to manage and maintain such infrastructure without additional support or training. Moreover, the initial costs associated with deploying and integrating a microservices-based system, including hardware upgrades and personnel training, could be a barrier for resource-constrained settings. However, the platform's architecture was designed to be open source which in turn might be an opportunity for medical education in low- and middle-income countries. By offering the framework of PATe 2.0, different faculties and universities are free to add their own sample collection and set their own focus in histopathological teaching.

## Conclusion

PATe 2.0 was developed through five iterative steps, each addressing critical aspects of its design and functionality. These iterations included analyzing and identifying limitations of the former PATe, designing a scalable microservices architecture and robust database, implementing a Worker API for computational tasks, developing a user-centric frontend, and optimizing performance with the Fast API. This iterative approach ensured scalability, usability, and responsiveness. Future updates will introduce features such as interactive quiz modes and advanced search tools, further enhancing its capabilities. With its modular design and innovative features, PATe 2.0 has the potential to bridge the gap between education, diagnostics, and research in modern pathology.

## Funding

This work received no external funding.

## CRediT authorship contribution statement

Conceptualization: C.B., G.R. Supervision: C.B. Resources: C.B. Writing: L.W., H.B. Software: H.B., A.A., V.R. Visualization: H.B. Critical review: C.B., G.R., J.A., B.R., A.A., V.R., L.W. All authors read and approved the final manuscript and had final responsibility for the decision to submit it for publication.

## Declaration of competing interest

The authors declare that they have no known competing financial interests or personal relationships that could have appeared to influence the work reported in this article.
